# Modified Backtracking Search Optimization Algorithm Inspired by Simulated Annealing for Constrained Engineering Optimization Problems

**DOI:** 10.1155/2018/9167414

**Published:** 2018-02-13

**Authors:** Hailong Wang, Zhongbo Hu, Yuqiu Sun, Qinghua Su, Xuewen Xia

**Affiliations:** ^1^School of Information and Mathematics, Yangtze University, Jingzhou, Hubei 434023, China; ^2^School of Software, East China Jiaotong University, Nanchang, Jiangxi 330013, China

## Abstract

The backtracking search optimization algorithm (BSA) is a population-based evolutionary algorithm for numerical optimization problems. BSA has a powerful global exploration capacity while its local exploitation capability is relatively poor. This affects the convergence speed of the algorithm. In this paper, we propose a modified BSA inspired by simulated annealing (BSAISA) to overcome the deficiency of BSA. In the BSAISA, the amplitude control factor (*F*) is modified based on the Metropolis criterion in simulated annealing. The redesigned *F* could be adaptively decreased as the number of iterations increases and it does not introduce extra parameters. A self-adaptive *ε*-constrained method is used to handle the strict constraints. We compared the performance of the proposed BSAISA with BSA and other well-known algorithms when solving thirteen constrained benchmarks and five engineering design problems. The simulation results demonstrated that BSAISA is more effective than BSA and more competitive with other well-known algorithms in terms of convergence speed.

## 1. Introduction

Optimization is an essential research objective in the fields of applied mathematics and computer sciences. Optimization algorithms mainly aim to obtain the global optimum for optimization problems. There are many different kinds of optimization problems in real world. When an optimization problem has a simple and explicit gradient information or requires relatively small budgets of allowed function evaluations, the implementation of classical optimization techniques such as mathematical programming often could achieve efficient results [1]. However, many real-world engineering optimization problems may have complex, nonlinear, or nondifferentiable forms, which make them difficult to be tackled by using classical optimization techniques. The emergence of metaheuristic algorithms has overcome the deficiencies of classical optimization techniques to some extent, as they do not require gradient information and have the ability to escape from local optima. Metaheuristic algorithms are mainly inspired from a variety of natural phenomena and/or biological social behavior. Among these metaheuristic algorithms, swarm intelligence algorithms and evolutionary algorithms perhaps are the most attractive [2]. Swarm intelligence algorithms [3] generally simulate the intelligence behavior of swarms of creatures, such as particle swarm optimization (PSO) [4], ant colony optimization (ACO) [5], cuckoo search (CS) [6], and the artificial bee colony (ABC) algorithm [7]. These types of algorithms generally are developed by inspirations from a series of complex behavior processes in swarms with mutual cooperation and self-organization, in which “cooperation” is their core concept. The evolutionary algorithms (EAs) [8, 9] are inspired by the mechanism of nature evolution, in which “evolution” is the key idea. Examples of EAs include genetic algorithm (GA) [10], differential evolution (DE) [11–14], covariance matrix adaptation evolution strategy (CMAES) [15], and the backtracking search optimization algorithm (BSA) [16].

BSA is an iterative population-based EA, which was first proposed by Civicioglu in 2013. BSA has three basic genetic operators: selection, mutation, and crossover. The main difference between BSA and other similar algorithms is that BSA possesses a memory for storing a population from a randomly chosen previous generation, which is used to generate the search-direction matrix for the next iteration. In addition, BSA has a simple structure, which makes it efficient, fast, and capable of solving multimodal problems. BSA has only one control parameter called the* mix-rate*, which significantly reduces the sensitivity of the initial values to the algorithm's parameters. Due to these characteristics, in less than 4 years, BSA has been employed successfully to solve various engineering optimization problems, such as power systems [17–19], induction motor [20, 21], antenna arrays [22, 23], digital image processing [24, 25], artificial neural networks [26–29], and energy and environmental management [30–32].

However, BSA has a weak local exploitation capacity and its convergence speed is relatively slow. Thus, many studies have attempted to improve the performance of BSA and some modifications of BSA have been proposed to overcome the deficiencies. From the perspective of modified object, the modifications of BSA can be divided into the following four categories. It is noted that we consider classifying the publication into the major modification category if it has more than one modification:

- Modifications of the initial populations [33–38]
- Modifications of the reproduction operators, including the mutation and crossover operators [39–47]
- Modifications of the selection operators, including the local exploitation strategy [48–51]
- Modifications of the control factor and parameter [52–57].

The research on controlling parameters of EAs is one of the most promising areas of research in evolutionary computation; even a little modification of parameters in an algorithm can make a considerable difference [58]. In the basic BSA, the value of amplitude control factor (*F*) is the product of three and the standard normal distribution random number (i.e., *F* = 3 · randn), which is often too large or too small according to its formulation. This may give BSA a powerful global exploration capability at the early iterations; however, it also affects the later exploitation capability of BSA. Based on these considerations, we focus mainly on the influence of the amplitude control factor (*F*) on the BSA, that is, the fourth of the categories defined above. Duan and Luo [52] redesigned an adaptive *F* based on the fitness statistics of population at each iteration. Wang et al. [53] and Tian et al. [54] proposed an adaptive *F* based on Maxwell-Boltzmann distribution. Askarzadeh and dos Santos Coelho [55] proposed an adaptive *F* based on Burger's chaotic map. Chen et al. [56] redesigned an adaptive *F* by introducing two extra parameters. Nama et al. [57] proposed a new *F* to adaptively change in the range of (0.45,1.99) and new mix-rate to randomly change in the range of (0,1). These modifications of *F* have achieved good effects in the BSA.

Different from the modifications of *F* in BSA described above, a modified version of BSA (BSAISA) inspired by simulated annealing (SA) is proposed in this paper. In the BSAISA, *F* based on iterations is redesigned by learning a characteristic where SA can probabilistically accept a higher energy state and the acceptance probability decreases with the decrease in temperature. The redesigned *F* can adaptively decrease as the number of iterations increases without introducing extra parameters. This adaptive variation tendency provides an efficient tradeoff between early exploration and later exploitation capability. We verified the effectiveness and competitiveness of BSAISA in simulation experiments using thirteen constrained benchmarks and five engineering design problems in terms of convergence speed.

The remainder of this paper is organized as follows.  Section 2 introduces the basic BSA. As the main contribution of this paper, a detailed explanation of BSAISA is presented in Section 3. In Section 4, we present two sets of simulation experiments in which we implemented BSAISA and BSA to solve thirteen constrained optimization and five engineering design problems. The results are compared with those obtained by other well-known algorithms in terms of the solution quality and function evaluations. Finally, we give our concluding remarks in Section 5.

## 2. Backtracking Search Optimization Algorithm (BSA)

BSA is a population-based iterative EA. BSA generates trial populations to take control of the amplitude of the search-direction matrix which provides a strong global exploration capability. BSA equiprobably uses two random crossover strategies to exchange the corresponding elements of individuals in populations and trial populations during the process of crossover. Moreover, BSA has two selection processes. One is used to select population from the current and historical populations; the other is used to select the optimal population. In general, BSA can be divided into five processes: initialization, selection I, mutation, crossover, and selection II [16].

### 2.1. Initialization

BSA generates initial population *P* and initial old population oldP using

(1) Pi,j~Ulowj,upj,oldPi,j~Ulowj,upj,

where *P*_*i*,*j*_ and oldP_*i*,*j*_ are the *j*th individual elements in the problem dimension (*D*) that falls in *i*th individual position in the population size (*N*), respectively, low_*j*_ and up_*j*_ mean the lower boundary and the upper boundary of the *j*th dimension, respectively, and *U* is a random uniform distribution.

### 2.2. Selection I

BSA's selection I process is the beginning of each iteration. It aims to reselect a new oldP for calculating the search direction based on population *P* and historical population oldP. The new oldP is reselected through the “if-then” rule in

(2) $$\begin{array}{c} \text{if }a<b\;\;then\;\;oldP≔P\;|\;a,b\sim U\left(\;0,1\;\right), \end{array}$$

where ≔ is the update operation; *a* and *b* represent random numbers between 0 and 1. The update operation (see (2)) ensures BSA has a memory. After oldP is reselected, the order of the individuals in oldP is randomly permuted by

(3) $$\begin{array}{c} oldP≔permuting\left(\;oldP\;\right). \end{array}$$

### 2.3. Mutation

The mutation operator is used for generating the initial form of trial population *M* with

(4) $$\begin{array}{c} M=P+F\cdot \left(\;oldP-P\;\right), \end{array}$$

where* F* is the amplitude control factor of mutation operator used to control the amplitude of the search direction. The value *F* = 3  ·  randn, where randn ~ *N*(0,1), and *N* is standard normal distribution.

### 2.4. Crossover

In this process, BSA generates the final form of trial population *T*. BSA equiprobably uses two crossover strategies to manipulate the selected elements of the individuals at each iteration. Both the strategies generate different binary integer-valued matrices (map) of size *N* · *D* to select the elements of individuals that have to be manipulated.

Strategy I uses the mix-rate parameter (mix-rate) to control the numbers of elements of individuals that are manipulated by using ⌈mix-rate · rand · *D*⌉, where mix-rate = 1. Strategy II manipulates the only one randomly selected element of individuals by using randi(*D*), where randi ~ *U*(0, *D*).

The two strategies equiprobably are employed to manipulate the elements of individuals through the “if-then” rule: if map_*n*,*m*_ = 1, where *n* ∈ {1,2,…, *N*} and *m* ∈ {1,2,…, *D*}, then, *T* is updated with *T*_*n*,*m*_≔*P*_*n*,*m*_.

At the end of crossover process, if some individuals in *T* have overflowed the allowed search space limits, they will need to be regenerated by using (1).

### 2.5. Selection II

In BSA's selection II process, the fitness values in *P*_*i*_ and *T*_*i*_ are compared, used to update the population *P* based on a greedy selection. If *T*_*i*_ have a better fitness value than *P*_*i*_, then, *T*_*i*_ is updated to be *P*_*i*_. The population *P* is updated by using

(5) $$\begin{array}{c} P_{i}=\left\{\begin{array}{cc} T_{i}, & \text{if fitness}\left(T_{i}\right)<\text{fitness}\left(P_{i}\right),\\ P_{i}, & \text{else}. \end{array}\right. \end{array}$$

If the best individual of *P* (*P*_best_) has a better fitness value than the global minimum value obtained, the global minimizer will be updated to be *P*_best_, and the global minimum value will be updated to be the fitness value of *P*_best_.

## 3. Modified BSA Inspired by SA (BSAISA)

As mentioned in the introduction of this paper, the research work on the control parameters of an algorithm is very meaningful and valuable. In this paper, in order to improve BSA's exploitation capability and convergence speed, we propose a modified version of BSA (BSAISA) where the redesign of *F* is inspired by SA. The modified details of BSAISA are described in this section. First, the structure of the modified *F* is described, before we explain the detailed design principle for the modified *F* inspired by SA. Subsequently, two numerical tests are used to illustrate that the redesigned *F* improves the convergence speed of the algorithm. We introduce a self-adaptive *ε*-constrained method for handling constraints at the end of this section.

### 3.1. Structure of the Adaptive Amplitude Control Factor *F*

The modified *F* is a normally distributed random number, where its mean value is an exponential function and its variance is equal to 1. In BSAISA, we redesign the adaptive *F* to replace the original version using

(6) $$\begin{array}{c} F_{i}\sim N\left(\;\exp\left(\;\frac{-1/\left|\Delta I_{i}\right|}{1/G}\;\right),1\;\right), \end{array}$$

where *i* is the index of individuals, *F*_*i*_ is the adaptive amplitude control factor that corresponds to the *i*th individual, |Δ*I*_*i*_| is the absolute value of the difference between the objective function values of *P*_*i*_ and *T*_*i*_ (individual differences), *N* is the normal distribution, and *G* is the current iteration.

According to (6), the exponential function (mean value) decreases dynamically with the change in the number of iterations (*G*) and the individual differences (|Δ*I*_*i*_|). Based on the probability density function curve of the normal distribution, the modified *F* can be decreased adaptively as the number of iterations increases. Another characteristic of the modified* F* is that there are not any extra parameters.

### 3.2. Design Principle of the Modified Amplitude Control Factor *F*

The design principle of the modified *F* is inspired by the Metropolis criterion in SA. SA is a metaheuristic optimization technique based on physical behavior in nature. SA based on the Monte Carlo method was first proposed by Metropolis et al. [59] and it was successfully introduced into the field of combinatorial optimization for solving complex optimization problems by Kirkpatrick et al. [60].

The basic concept of SA derives from the process of physical annealing with solids. An annealing process occurs when a metal is heated to a molten state with a high temperature; then it is cooled slowly. If the temperature is decreased quickly, the resulting crystal will have many defects and it is just metastable; even the most stable crystalline state will be achieved at all. In other words, this may form a higher energy state than the most stable crystalline state. Therefore, in order to reach the absolute minimum energy state, the temperature needs to be decreased at a slow rate. SA simulates this process of annealing to search the global optimal solution in an optimization problem. However, accepting only the moves that lower the energy of system is like extremely rapid quenching; thus SA uses a special and effective acceptance method, that is, Metropolis criterion, which can probabilistically accept the hill-climbing moves (the higher energy moves). As a result, the energy of the system evolves into a Boltzmann distribution during the process of the simulated annealing. From this angle of view, it is no exaggeration to say that the Metropolis criterion is the core of SA.

The Metropolis criterion can be expressed by the physical significance of energy, where the new energy state will be accepted when the new energy state is lower than the previous energy state, and the new energy state will be probabilistically accepted when the new energy state is higher than the previous energy state. This feature of SA can escape from being trapped in local minima especially in the early stages of the search. It can also be described as follows.

(i) If  Δ*E* = *E*_*j*_ − *E*_*i*_ ≤ 0, then the new state *j* is accepted and the energy with the displaced atom is used as the starting point for the next step, where *E* represents the energy of the atom. Both *i* and *j* are the states of atoms, and *j* is the next state of *i*.

(ii) If  Δ*E* > 0, then calculate the probability of *P*(Δ*E*) = exp⁡(−Δ*E*/*kT*_*c*_), and generate a random number *θ*, which is a uniform distribution over (0,1), where *k* is Boltzmann's constant (in general, *k* = 1) and *T*_*c*_ is the current temperature. If *P*(Δ*E*) > *θ*, then the new energy will be accepted; otherwise, the previous energy is used to start the next step.

**Analysis 1 .**

The Metropolis criterion states that SA has two characteristics: (1) SA can probabilistically accept the higher energy and (2) the acceptance probability of SA decreases as the temperature decreases. Therefore, SA can reject and jump out of a local minimum with a dynamic and decreasing probability to continue exploiting the other solutions in the state space. This acceptance mechanism can enrich the diversity of energy states.

**Analysis 2 .**

As shown in (4), *F* is used to control the amplitude of population mutation in BSA, thus *F* is an important factor for controlling population diversity. If *F* is excessively large, the diversity of the population will be too high and the convergence speed of BSA will slow down. If *F* is excessively small, the diversity of the population will be reduced so it will be difficult for BSA to obtain the global optimum and it may be readily trapped by a local optimum. Therefore, adaptively controlling the amplitude of *F* is a key to accelerating the convergence speed of the algorithm and maintaining its the population diversity.

Based on Analyses 1 and 2, it is clear that if *F* can dynamically decrease, the convergence speed of BSA will be able to accelerate while maintaining the population diversity. On the other hand, SA possesses this characteristic that its acceptance probability can be dynamically reduced. Based on these two considerations, we propose BSAISA with a redesigned *F*, which is inspired by SA. More specifically, the new *F* (see (6)) is redesigned by learning the formulation (*P*(Δ*E*)) of acceptance probability, and its formulation has been shown in the previous subsection.

For the two formulas of the modified *F* and *P*(Δ*E*), the individual difference (|Δ*I*_*i*_|) of a population or the energy difference (Δ*E*) of a system will decrease as the number of iterations increases in an algorithm, and the temperature of SA tends to decrease, while the iteration of BSA tends to increase. As a result, one can observe the correspondence between modified *F* and *P*(Δ*E*) where the reciprocal of individual difference (1/|Δ*I*_*i*_|) corresponds to the energy difference (Δ*E*) of SA, and the reciprocal of current iteration (1/*G*) corresponds to the current temperature (*T*_*c*_) of SA. In this way, the redesigned *F* can be decreased adaptively as the number of iterations increases.

### 3.3. Numerical Analysis of the Modified Amplitude Control Factor *F*

**Figure 1:**
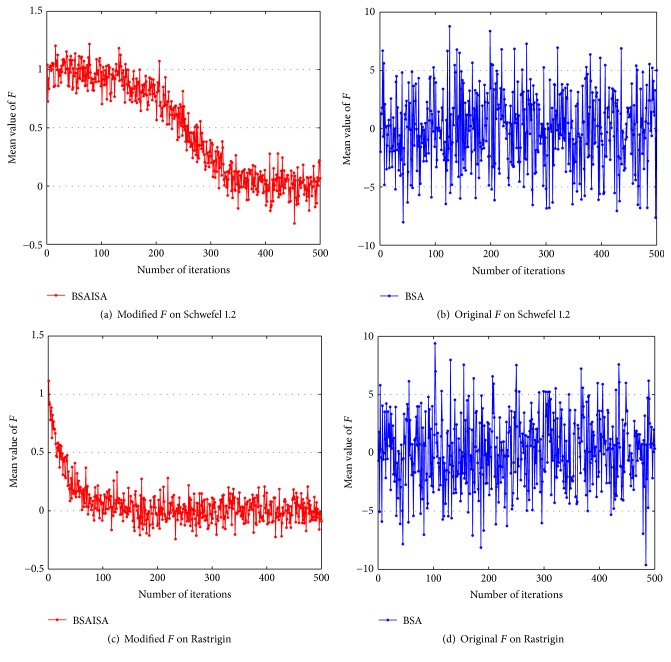
Comparisons of modified *F* and original *F* for Schwefel 1.2 and Rastrigin.

**Figure 2:**
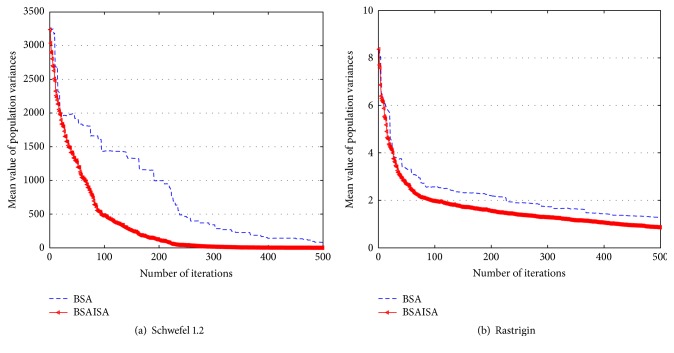
The mean values of population variances versus number of iterations using BSA and BSAISA for two functions.

**Figure 3:**
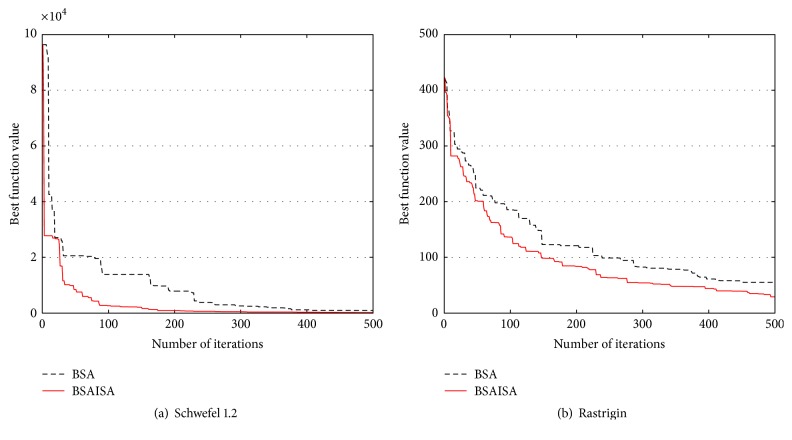
Convergence curves of BSA and BSAISA for two functions.

In order to verify that the convergence speed of the basic BSA is improved with the modified *F*, two types (unimodal and multimodal) of unconstrained benchmark functions are used to test the changing trends in *F* and population variances and best function values as the iterations increases, respectively. The two functions are Schwefel 1.2 and Rastrigin, and their detailed information is provided in [61]. The two functions and the user parameters including the populations (*N*), dimensions (*D*), and maximum iterations (Max) are shown in Table 1. Three groups of test results are compared in the tests including (1) the comparative curves of the mean values of the modified *F* and original *F* for Schwefel 1.2 and Rastrigin, (2) the comparative curves of the mean values of BSA and BSAISA population variances for two functions, and (3) the convergence curves of BSA and BSAISA for two functions. They are depicted in Figures 1, 2, and 3, respectively. Based on Figures 1–3, two results can be observed as follows.

(1) According to the trends of* F* in Figure 1, both the original* F* and modified* F* are subject to changes from the normal distribution. The mean value of the original* F* does not tend to decrease as the number of iterations increases. By contrast, the mean value of the modified* F* exhibits a clear and fluctuating downward trend as the number of iterations increases.

(2) According to Figure 2, the population variances of BSAISA and BSA both exhibit a clear downward trend as the number of iterations increases. The population variances of BSAISA and BSA are almost same during the early iterations. This illustrates that the modified* F* does not reduce the population diversity in the early iterations. In the middle and later iterations, the population variances of BSAISA decrease more quickly than that of BSA. This illustrates that the modified* F* improves the convergence speed. As can be seen from Figure 3, as the number of iterations increases, the best objective function value of BSAISA drops faster than that of BSA. This shows that the modified* F* improves the convergence speed of BSA. Moreover, BSAISA can find a more accurate solution at the same computational cost.

*Summary*. Based on the design principle and numerical analysis of the modified *F*, the modified *F* exhibits an overall fluctuating and downward trend, which matches with the concept of the acceptance probability in SA. In particular, during the early iterations, the modified *F* is relatively large. This allows BSAISA to search in a wide region, while maintaining the population diversity of BSAISA. As the number of iterations decreases, the modified *F* gradually exhibits a decreasing trend. This accelerates the convergence speed of BSAISA. In the later iterations, the modified *F* is relatively small. This enables BSAISA to fully search in the local region. Therefore, it can be concluded that BSAISA can adaptively control the amplitude of population mutation to change its local exploitation capacity. This may improve the convergence speed of the algorithm. Moreover, the modified *F* does not introduce extra parameters, so it does not increase the sensitivity of BSAISA to the parameters.

### 3.4. A Self-Adaptive *ε*-Constrained Method for Handling Constraints

In general, a constrained optimization problem can be mathematically formulated as a minimization problem, as follows:

(7) min fXsubject  to: giX≤0,i=1,2,…,m, hjX=0,j=1,2,…,n,

where *X* = (*x*_1_, *x*_2_,…, *x*_*D*_) ∈ *R*^*D*^ is a *D*-dimensional vector, *m* is the total number of inequality constraints, and* n* is the total number of equality constraints. The equality constraints are transformed into inequality constraints by using |*h*_*j*_(*X*)|−*δ* ≤ 0, where *δ* is a very small degree of violation, and *δ* = 1*E* − 4 in this paper. The maximization problems are transformed into minimization problems using −*f*(*X*). The constraint violation *φ*(*X*) is given by

(8) $$\begin{array}{c} \varphi \left(\;X\;\right)=\overset{m}{\underset{i=1}{\sum }}\max\left(\;0,g_{i}\left(\;X\;\right)\;\right)+\overset{n}{\underset{j=1}{\sum }}\max\left(\;0,\left|\;h_{j}\left(\;X\;\right)\;\right|-\delta \;\right). \end{array}$$

Several constraint-handling methods have been proposed previously, where the five most commonly used methods comprise penalty functions, feasibility and dominance rules (FAD), stochastic ranking, *ε*-constrained methods, and multiobjectives concepts. Among these five methods, the *ε*-constrained method is relatively effective and used widely. Zhang et al. [62] proposed a self-adaptive *ε*-constrained method (SA*ε*) to combine with the basic BSA for constrained problems. It has been verified that the SA*ε* has a stronger search efficiency and convergence than the fixed *ε*-constrained method and FAD. In this paper, the SA*ε* is used to combine with BSAISA for constrained optimization problems, which comprises the following two rules: (1) if the constraint violations of two solutions are smaller than a given *ε* value or two solutions have the same constraint violations, the solution with a better objective function value is preferred and (2) if not, the solution with a smaller constraint violation is preferred. SA*ε* could be expressed by the following equations:

(9) $$\begin{array}{c} \begin{array}{c} the\;\;better\;\;one=f\left(X_{1}\right)\left\{\begin{array}{cc} f\left(X_{1}\right)<f\left(X_{2}\right), & \text{if}\;\;\varphi \left(X_{1}\right),\;\;\varphi \left(X_{2}\right)\leq \varepsilon ,\\ f\left(X_{1}\right)<f\left(X_{2}\right), & \text{if}\;\;\varphi \left(X_{1}\right)=\varphi \left(X_{2}\right),\\ \varphi \left(X_{1}\right)\leq \varphi \left(X_{2}\right), & otherwise, \end{array}\right. \end{array} \end{array}$$

where *ε* is a positive value that represents a tolerance related to constraint violation. The self-adaptive *ε* value is formulated as the following equation:

(10) ε0=φP0θ,

(11) ε10=ε0,

(12) ε2t=φTtθ,if ε0>Th1,ε0,else,

(13) ε1t=ε2t,if ε2t>Th2,  ε2t<ε1t−1,ε1t−1,else,

(14) εt=ε1t1−tTccp,if  t≤Tc0,else,

where *t* is the number of the current iterations. *φ*(*P*_0_^*θ*^) is the constraint violation of the top *θ*th individual in the initial population. *φ*(*T*_*t*_^*θ*^) is the constraint violation of the top *θ*th individual in the trial population at the current iteration. cp and *T*_*c*_ are control parameters. Th1 and Th2 are threshold values. *εt* is related to the iteration *t* and functions *ε*_1_(*t*) and *ε*_2_(*t*).

Firstly, *ε*_0_ is set as *φ*(*P*_0_^*θ*^). If the initial value *ε*_0_ is bigger than Th1, *φ*(*T*_*t*_^*θ*^) will be assigned to *ε*_2_(*t*); otherwise *ε*_0_ will be assigned to *ε*_2_(*t*). Then, if *ε*_2_(*t*) < *ε*_1_(*t* − 1) and *ε*_2_(*t*) is bigger than Th2, *ε*_2_(*t*) will be assigned to *ε*_1_(*t*); otherwise *ε*_1_(*t* − 1) will be assigned to *ε*_1_(*t*). Finally, *εt* is updated as (14). The detailed information of SA*ε* can be acquired from [62], and the related parameter settings of SA*ε* (the same as [62]) are presented in Table 2.

**Figure 4:**
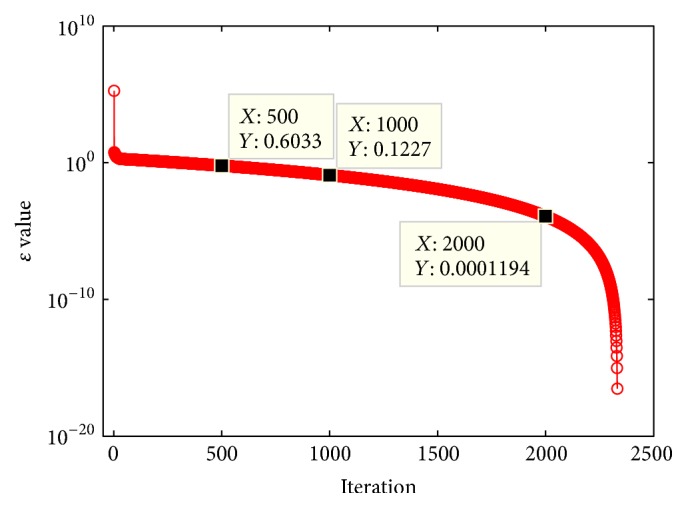
Plot of *ε* value with iteration.

To illustrate the changing trend of the self-adaptive *ε* value vividly, BSAISA with SA*ε* is used to solve a well-known benchmark constrained function G10 in [61]. The related parameters are set as *N* = 30, *θ* = 0.3*N*, cp = 5, *T*_*c*_ = 2333, Th1 = 10, and Th2 = 2. The changing trend of *ε* value is shown in Figure 4. Three sampling points, that is, *ε*(500) = 0.6033, *ε*(1000) = 0.1227, and *ε*(2000) = 1.194*E* − 4, are marked in Figure 4. As shown in Figure 4, it can be observed that *ε* value declines very fast at first. After it is smaller than about 2, it declines as an exponential way. This changing trend of *ε* value could help algorithm to sufficiently search infeasible domains near feasible domains.

The pseudocode for BSAISA is showed in Pseudocode 1. In Pseudocode 1, the modified adaptive *F* is shown in lines (14)–(16). When BSAISA deals with constrained optimization problems, the code in line (8) and line (40) in Pseudocode 1 should consider objective function value and constraint violation simultaneously, and SA*ε* is applied to choose a better solution or best solution in line (42) and lines (47)-(48).

## 4. Experimental Studies

In this section, two sets of simulation experiments were executed to evaluate the effectiveness of the proposed BSAISA. The first experiment set performed on 13 well-known benchmark constrained functions taken from [63] (see Appendix A). These thirteen benchmarks contain different properties as shown in Table 3, including the number of variables (*D*), objective function types, the feasibility ratio (*ρ*), constraint types and number, and the number of active constraints in the optimum solution. The second experiment is conducted on 5 engineering constrained optimization problems chosen from [64] (see Appendix B). These five problems are the three-bar truss design problem (TTP), pressure vessel design problem (PVP), tension/compression spring design problem (TCSP), welded beam design problem (WBP), and speed reducer design problem (SRP), respectively. These engineering problems include objective functions and constraints of various types and natures (quadratic, cubic, polynomial, and nonlinear) with various number of design variables (continuous, integer, mixed, and discrete).

The recorded experimental results include the best function value (Best), the worst function value (Worst), the mean function value (Mean), the standard deviation (Std), the best solution (variables of best function value), the corresponding constraint value, and the number of function evaluations (FEs). The number of function evaluations can be considered as a convergence rate or a computational cost.

In order to evaluate the performance of BSAISA in terms of convergence speed, the FEs are considered as the best FEs corresponding to the obtained best solution in this paper. The calculation of FEs are the product of population sizes (*N*) and the number of iterations (Ibest) at which the best function value is first obtained (i.e., FEs = *N∗*Ibest). For example, if 2500 is the maximum number of iterations for one minimization problem, *f*(1999) = 0.0126653, *f*(2000) = 0.0126652, *f*(2500) = *f*(2000) = 0.0126652, the Ibest value should be 2000. However, BSAISA needs to evaluate the initial historical population (oldP), so its actual FEs should be plus *N* (i.e., FEs = *N∗*Ibest + *N*).

### 4.1. Parameter Settings

For the first experiment, the main parameters for 13 benchmark constrained functions are the same as the following: population size (*N*) is set as 30; the maximum number of iterations (*T*_max_) is set as 11665. Therefore, BSAISA's maximum number of function evaluations (MFEs) should equal to 34,9980 (nearly 35,0000). The 13 benchmarks were executed by using 30 independent runs.

For the 5 real-world engineering design problems, we use slightly different parameter settings since each problem has different natures, that is, TTP (*N* = 20, *T*_max_ = 1000), PVP (*N* = 20, *T*_max_ = 3000), TCSP (*N* = 20, *T*_max_ = 3000), WBP (*N* = 20, *T*_max_ = 3000), SRP (*N* = 20, *T*_max_ = 2000). The 6 engineering problems were performed using 50 independent runs.

The user parameters of all experiments are presented in Table 4. Termination condition may be the maximum number of iterations, CPU time, or an allowable tolerance value between the last result and the best known function value for most metaheuristics. In this paper, the maximum number of iterations is considered as termination condition. All experiments were conducted on a computer with a Intel(R) Core(TM) i5-4590 CPU @ 3.30 GHz and 4 GB RAM.

### 4.2. Simulation on Constrained Benchmark Problems

In this section, BSAISA and BSA are performed on the 13 benchmarks simultaneously. Their statistical results obtained from 30 independent runs are listed in Tables 5 and 6, including the best known function value (Best Known) and the obtained Best/Mean/Worst/Std values as well as the FEs. The best known values for all the test problems are derived from [62]. The best known values found by algorithms are highlighted in* bold*. From Tables 5 and 6, it can be seen that BSAISA is able to find the known optimal function values for G01, G04, G05, G06, G08, G09, G011, G012, and G013; however, BSA fails to find the best known function value on G09 and G13. For the rest of the functions, BSAISA obtains the results very close to the best known function values. Moreover, BSAISA requires fewer FEs than BSA on G01, G03, G04, G05, G06, G07, G08, G09, G011, and G012. Although the FEs of BSAISA are slightly worse than that of BSA for G02, G10, and G13, BSAISA finds more accurate best function values than BSA for G02, G03, G07, G09, G10, and G13.

**Figure 5:**
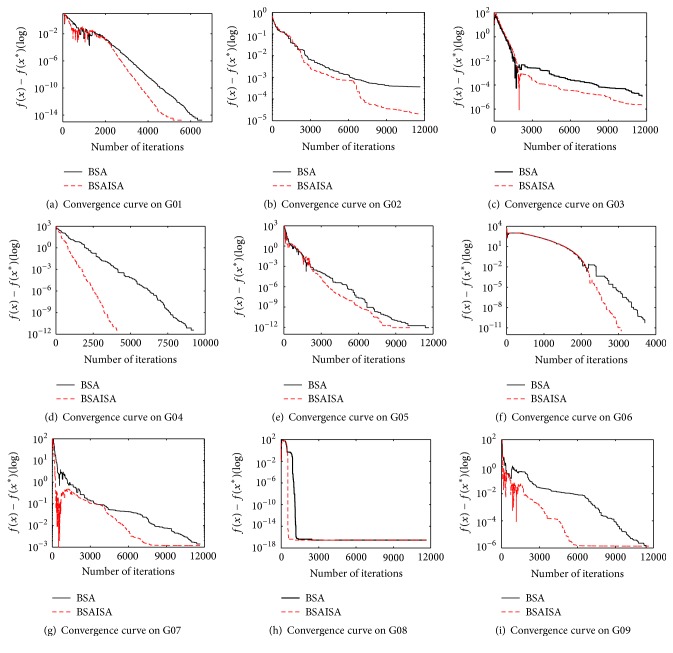
The convergence curves of the first 9 functions by BSAISA and BSA.

**Figure 6:**
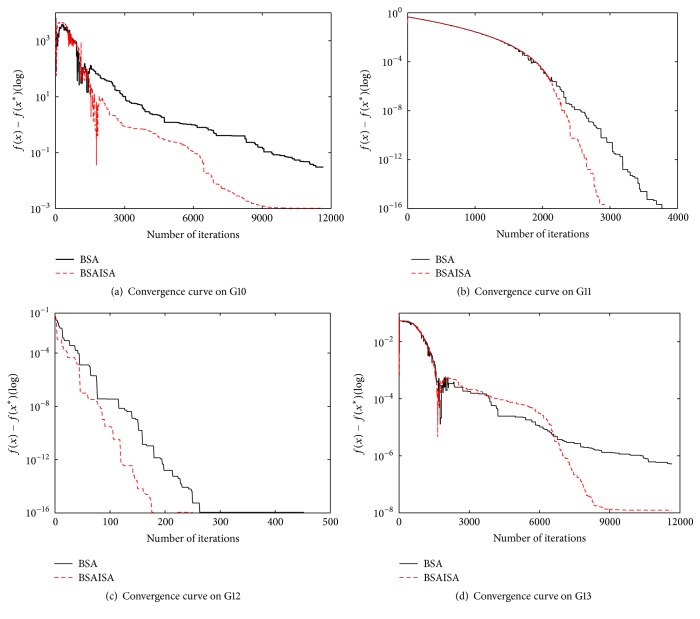
The convergence curves of the latter 4 functions by BSAISA and BSA.

To further compare BSAISA and BSA, the function value convergence curves of 13 functions that have significant differences have been plotted, as shown in Figures 5 and 6. The horizontal axis represents the number of iterations, while the vertical axis represents the difference of the objective function value and the best known value. For G02, G04, G06, G08, G11, and G12, it is obvious that the convergence speed of BSAISA is faster than that of BSA, and its best function value is also better than that of BSA. For G01, G03, G05, G07, G09, G10, and G13, it can be observed that the objective function values of BSAISA and BSA fluctuate in the early iterations, and they decrease as the number of iterations increases during the middle and late stages. This illustrates that both the algorithms are able to escape the local optimum under different degrees, and the convergence curve of BSAISA drops still faster than that of BSA. Therefore, the experiment results demonstrate that the convergence speed of the basic BSA is improved with our modified *F*.

In order to further verify the competitiveness of BSAISA in aspect of convergence speed, we compared BSAISA with some classic and state-of-the-art approaches in terms of best function value and function evaluations. The best function value and the corresponding FEs of each algorithm on 13 benchmarks are presented in Table 7, where the optimal results are in* bold* on each function. These compared algorithms are listed below:

1. Stochastic ranking (SR) [63]
2. Filter simulated annealing (FSA) [65]
3. Cultured differential evolution (CDE) [66]
4. Agent based memetic algorithm (AMA) [64]
5. Modified artificial bee colony (MABC) algorithm [67]
6. Rough penalty genetic algorithm (RPGA) [68]
7. BSA combined self-adaptive *ε* constrained method (BSA-SA*ε*) [62].

To compare these algorithms synthetically, a simple evaluation mechanism is used. It can be explained as the best function value (Best) is preferred, and the function evaluations (FEs) are secondary. More specifically, (1) if one algorithm has a better* Best* than those of others on a function, there is no need to consider* FEs* and the algorithm is superior to other algorithms on this function. (2) If two or more algorithms have found the optimal* Best* on a function, the algorithm with the lowest* FEs* is considered as the winner on the function. (3) Record the number of winners and the number of the optimal function values for each algorithm on the set of benchmarks, and then give the sort for all algorithms.

From Table 7, it can be observed that the rank of these 8 algorithms is as follows: BSAISA, CDE, BSA-SA*ε*, MABC, SR, RPGA, FSA, and AMA. Among the 13 benchmarks, BSAISA wins on 6 functions and it is able to find the optimal values of 10 functions. This is better than all other algorithms, thus BSAISA ranks the first. The second algorithm CDE performs better on G02, G07, G09, and G10 than BSAISA but worse on G01, G03, G08, G11, G12, and G13. BSA-SA*ε* obtains the optimal function values of 10 functions but requires more function evaluations than BSAISA and CDE, so it should rank the third. MABC ranks the fourth. It obtains the optimal function values of 7 functions, which are fewer in number than those of the former three algorithms. Both SR and RPGA have found the same number of the optimal function values, while the former is the winner on G04, so SR is slightly better than RPGA. As for the last two algorithms, FSA and AMA just perform well on three functions, while FSA is the winner on G06, so FSA is slightly better than AMA.

Based on the above comparison, it can be concluded that BSAISA is effective and competitive in terms of convergence speed.

### 4.3. Simulation on Engineering Design Problems

In order to assess the optimization performance of BSAISA in real-world engineering constrained optimization problems, 5 well-known engineering constrained design problems including three-bar truss design, pressure vessel design, tension/compression spring design, welded beam design, and speed reducer design are considered in the second experiment.

#### 4.3.1. Three-Bar Truss Design Problem (TTP)

The three-bar truss problem is one of the engineering minimization test problems for constrained algorithms. The best feasible solution is obtained by BSAISA at *x* = (0.788675, 0.408248) with the objective function value *f*(*x*) = 263.895843 using 8940 FEs. The comparison of the best solutions obtained from BSAISA, BSA, differential evolution with dynamic stochastic selection (DEDS) [69], hybrid evolutionary algorithm (HEAA) [70], hybrid particle swarm optimization with differential evolution (POS-DE) [71], differential evolution with level comparison (DELC) [72], and mine blast algorithm (MBA) [73] is presented in Table 8. Their statistical results are listed in Table 9.

From Tables 8 and 9, BSAISA, BSA, DEDS, HEAA, PSO-DE, and DELC all reach the best solution with the corresponding function value equal to 263.895843 except MBA with 263.895852. However, BSAISA requires the lowest FEs (only 8940) among all algorithms. Its Std value is better than BSA, DEDS, HEAA, PSO-DE, and MBA except DELC. These comparative results indicate that BSAISA outperforms other algorithms in terms of computational cost and robustness for this problem.

**Figure 7:**
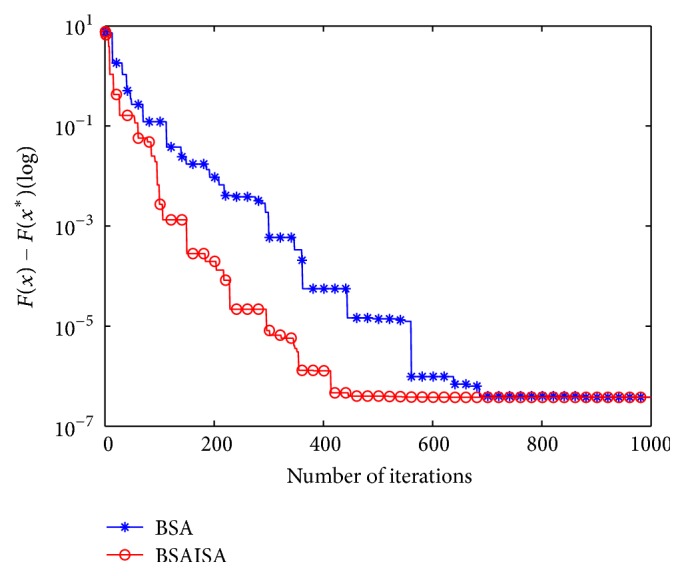
Convergence curves of BSAISA and BSA for the three-bar truss design problem.

Figure 7 depicts the convergence curves of BSAISA and BSA for the three-bar truss design problem, where the value of *F*(*x*^*∗*^) on the vertical axis equals 263.895843. As shown in Figure 7, BSA achieves the global optimum at about 700 iterations, while BSAISA only reaches the global optimum at about 400 iterations. It can be concluded that the convergence speed of BSAISA is faster than that of BSA for this problem.

#### 4.3.2. Pressure Vessel Design Problem (PVP)

The pressure vessel design problem has a nonlinear objective function with three linear and one nonlinear inequality constraints and two discrete and two continuous design variables. The values of the two discrete variables (*x*_1_, *x*_2_) should be the integer multiples of 0.0625. The best feasible solution is obtained by BSAISA at *x* = (0.8750, 0.4375, 42.0984, 176.6366) with the objective function value *f*(*x*) = 6059.7143 using 31,960 FEs.

For this problem, BSAISA is compared with nine algorithms: BSA, BSA-SA*ε* [62], DELC, POS-DE, genetic algorithms based on dominance tournament selection (GA-DT) [73], modified differential evolution (MDE) [74], coevolutionary particle swarm optimization (CPSO) [75], hybrid particle swarm optimization (HPSO) [76], and artificial bee colony algorithm (ABC) [77]. The comparison of the best solutions obtained by BSAISA and other reported algorithms is presented in Table 10. The statistical results of various algorithms are listed in Table 11.

As shown Table 10, the obtained solution sets of all algorithms satisfy the constraints for this problem. BSAISA, BSA-SA*ε*, ABC, DELC, and HPSO find the same considerable good objective function value 6059.7143, which is slightly worse than MDE's function value 6059.7143. It is worth mentioning that MBA's best solution was obtained at *x* = (0.7802, 0.3856, 40.4292, 198.4964) with *f*(*x*) = 5889.3216 and the corresponding constraint values equal to *g*_*i*_(*x*) = (0, 0, −86.3645, −41.5035) in [78]. Though MBA finds a far better function value than that of MDE, its obtained variables (i.e., 0.7802 and 0.3856) are not integer multiples of 0.0625. So they are not listed in Table 10 to ensure a fair comparison. From Table 11, except for MDE with the function value of 6059.7016, BSAISA offers better function value results compared to GA-DT, CPSO, ABC, and BSA. Besides that, BSAISA is far superior to other algorithms in terms of FEs. Unfortunately, the obtained Std value of BSAISA is relatively poor compared with others for this problem.

**Figure 8:**
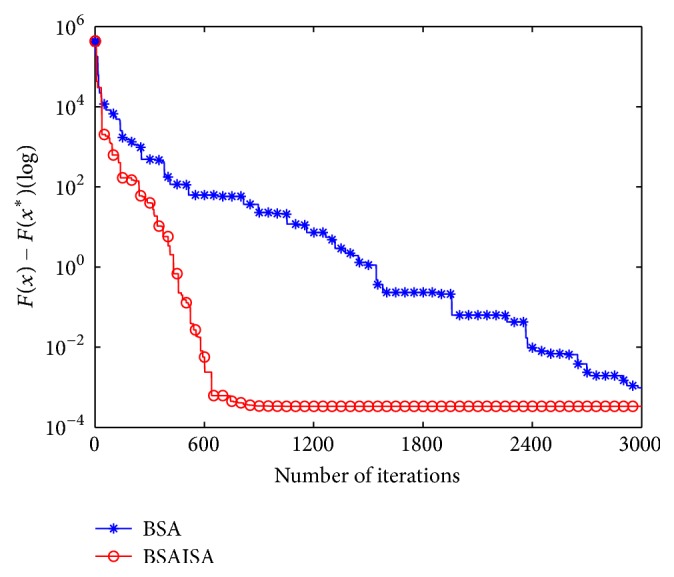
Convergence curves of BSAISA and BSA for the pressure vessel design problem.

Figure 8 describes the convergence curves of BSAISA and BSA for the pressure vessel design problem, where the value of *F*(*x*^*∗*^) on the vertical axis equals 6059.7143. As shown in Figure 8, BSAISA is able to find the global optimum at about 800 iterations and obtains a far more accurate function value than that of BSA. Moreover, the convergence speed of BSAISA is much faster than that of BSA.

#### 4.3.3. Tension Compression Spring Design Problem (TCSP)

This design optimization problem has three continuous variables and four nonlinear inequality constraints. The best feasible solution is obtained by BSAISA at *x* = (0.051687, 0.356669, 11.291824) with *f*(*x*) = 0.012665 using 9440 FEs. This problem has been solved by other methods as follows: GA-DT, MDE, CPSO, HPSO, DEDS, HEAA, DELC, POS-DE, ABC, MBA, BSA-SA*ε*, and Social Spider Optimization (SSOC) [79]. The comparison of the best solutions obtained from various algorithms is presented in Table 12. Their statistical results are listed in Table 13.

From Tables 12 and 13, the vast majority of algorithms can find the best function value 0.012665 for this problem, while GA-DT and CPSO fail to find it. With regard to the computational cost (FEs), BSAISA only requires 9440 FEs when it reaches the global optimum, which is superior to all other algorithms except MBA with 7650 FEs. However, the Worst and Mean and Std values of BSAISA are better than those of MBA. Consequently, for this problem, it can be concluded that BSA has the obvious superiority in terms of FEs over all other algorithms except MBA. Moreover, BSAISA has a stronger robustness when compared with MBA alone.

**Figure 9:**
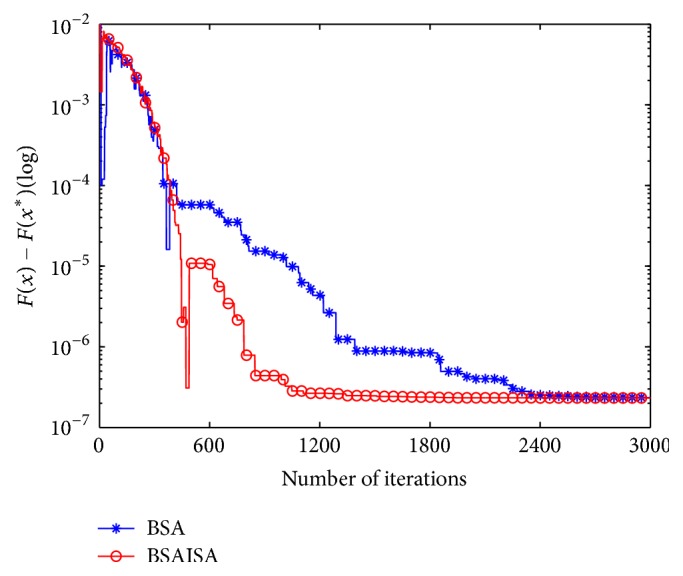
Convergence curves of BSAISA and BSA for the tension compression spring design problem.

Figure 9 depicts the convergence curves of BSAISA and BSA for the tension compression spring design problem, where the value of *F*(*x*^*∗*^) on the vertical axis equals 0.012665. From Figure 9 it can be observed that both BSAISA and BSA fall into a local optimum in the early iterations but they are able to successfully escape from the local optimum. However, the convergence speed of BSAISA is obviously faster than that of BSA.

#### 4.3.4. Welded Beam Design Problem (WBP)

The welded beam problem is a minimum cost problem with four continuous design variables and subject to two linear and five nonlinear inequality constraints. The best feasible solution is obtained by BSAISA at *x* = (0.205730, 3.470489, 9.036624, 0.205730) with the objective function value *f*(*x*) = 1.724852 using 29,000 FEs.

For this problem, BSAISA is compared with many well-known algorithms as follows: GA-DT, MDE, CPSO, HPSO, DELC, POS-DE, ABC, MBA, BSA, BSA-SA*ε*, and SSOC. The best solutions obtained from BSAISA and other well-known algorithms are listed in Table 14. The comparison of their statistical results is presented in Table 15.

From Tables 14 and 15, except that the constraint value of PSO is not available, the obtained solution sets of all algorithms satisfy the constraints for the problem. Most of algorithms including BSAISA, BSA, BSA-SA*ε*, MDE, HPSO, DELC, POS-DE, ABC, and SSOC are able to find the best function value 1.724852, while GA-DT and CPSO and MBA fail to find it. It should be admitted that DELC is superior to all other algorithms in terms of FEs and robustness for this problem. On the other hand, except for DELC, MDE, and SSOC with FEs of 2000, 2400, and 2500, respectively, BSAISA requires fewer FEs than the remaining algorithms (excluding algorithms that do not reach the best solution). When considering the comparison of the Std values for this problem, MBA exhibits its powerful robustness and BSAISA performs better than most algorithms except MBA, DELC, PSO-DE, BSA, and BSA-SA*ε*.

It is worth mentioning that from [74] the Worst, Mean, Best, and Std value of MDE are given as 1.724854, 1.724853, 1.724852, and 1.0*E* − 15, respectively. However, the corresponding values of DELC equal 1.724852, 1.724852, 1.724852, and 4.1*E* − 13, respectively, where its Worst and Mean values are smaller than those of MDE while its Std is bigger than those of MDE. So we consider that the Std of MDE is probably an error data for this problem, and we replace it with NA in Table 15.

**Figure 10:**
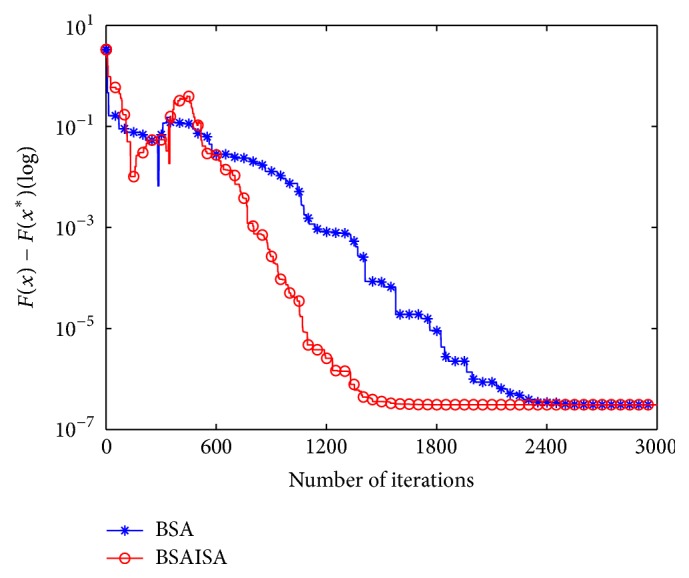
Convergence curves of BSAISA and BSA for the welded beam design problem.

Figure 10 depicts the convergence curves of BSAISA and BSA for the welded beam design problem, where the value of *F*(*x*^*∗*^) on the vertical axis equals 1.724852. Figure 10 shows the convergence speed of BSAISA is faster than that of BSA remarkably.

#### 4.3.5. Speed Reducer Design Problem (SRP)

This speed reducer design problem has eleven constraints and six continuous design variables (*x*_1_, *x*_2_, *x*_4_, *x*_5_, *x*_6_, *x*_7_) and one integer variable (*x*_3_). The best solution obtained from BSAISA is *x* = (3.500000, 0.7000000, 17, 7.300000, 7.715320, 3.350215, 5.286654) with *f*(*x*) = 2994.471066 using 15,860 FEs. The comparison of the best solutions obtained by BSAISA and other well-known algorithms is given in Table 16. The statistical results of BSAISA, BSA, MDE, DEDS, HEAA, DELC, POS-DE, ABC, MBA, and SSOC are listed in Table 17.

As shown in Tables 16 and 17, the obtained solution sets of all algorithms satisfy the constraints for this problem. BSAISA, BSA, DEDS, and DELC are able to find the best function value 2994.471066 while the others do not. Among the four algorithms, DEDS, DELC, and BSA require 30000, 30000, and 25640 FEs, respectively. However, BSAISA requires only 15,860 FEs when it reaches the same best function value. MBA fails to find the best known function value; thus BSAISA is better than MBA in this problem, even though MBA has lower FEs. As for the comparison of the Std, among the four algorithms that achieve the best known function value, BSAISA is worse than the others. However, one thing that should be mentioned is that the main purpose of the experiment is to compare the convergence speed between BSAISA and other algorithms. From this point of view, it can be concluded that BSAISA has a better performance than other algorithms in terms of convergence speed.

**Figure 11:**
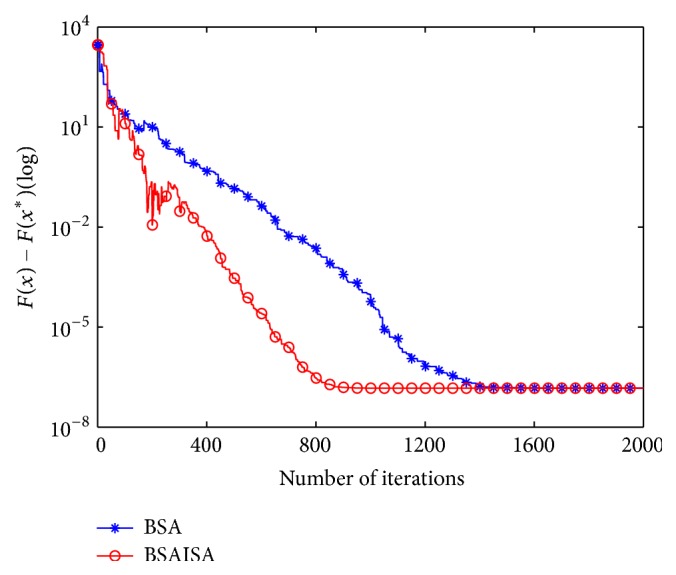
Convergence curves of BSAISA and BSA for the speed reducer design problem.

Figure 11 depicts the convergence curves of BSAISA and BSA for the speed reducer design problem, where the value of *F*(*x*^*∗*^) on the vertical axis equals 2994.471066. Figure 11 shows that the convergence speed of BSAISA is faster than that of BSA.

### 4.4. Comparisons Using Sign Test

Sign Test [80] is one of the most popular statistical methods used to determine whether two algorithms are significantly different. Recently, Miao et al. [81] utilized Sign Test method to analyze the performances between their proposed modified algorithm and the original one. In this paper, the two-tailed Sign Test with a significance level 0.05 is adopted to test the significant differences between the results obtained by different algorithms, and the test results are given in Table 18. The values of Best and FEs are two most important criterions for the evaluations of algorithms in our paper; they therefore should be chosen as the objectives of the Sign Test. The signs “+,” “≈,” and “−” represent, respectively, the fact that our BSAISA performs significantly better than, almost the same as, or significantly worse than the algorithm it is compared to. The null hypothesis herein is that the performances between BSAISA and one of the others are not significantly differential.

As shown in Table 18, the *p* values of supporting the null hypothesis of Sign Test for six pairs of algorithms (BSAISA-SR, BSAISA-FSA, BSAISA-AMA, BSAISA-MABC, BSAISA-RPGA, and BSAISA-BSA-SA*ε*) are 0.006, 0.003, 0.000, 0.012, 0.001, and 0.039, respectively, and thereby we can reject the null hypothesis. This illustrates that the optimization performance of the proposed BSAISA is significantly better than those of the six algorithms. The *p* value of BSAISA-CDE is equal to 0.581, which shows that we cannot reject the null hypothesis. However, according to the related sign values (“+,” “≈,” and “−”) from Table 18, BSAISA is slightly worse than CDE on 5 problems but wins on another 8 problems, which illustrates that the proposed BSAISA has a relatively excellent competitiveness compared with the CDE. Generally, the statistical *p* values and sign values validate that BSAISA has the superiority compared to the other well-known algorithms on the constrained optimization problems.

On the one hand, all experimental results suggest that the proposed method improves the convergence speed of BSA. On the other hand, the overall comparative results of BSAISA and other well-known algorithms demonstrate that BSAISA is more effective and competitive for constrained and engineering optimization problems in terms of convergence speed.

## 5. Conclusions and Future Work

In this paper, we proposed a modified version of BSA inspired by the Metropolis criterion in SA (BSAISA). The Metropolis criterion may probabilistically accept a higher energy state and the acceptance probability can decrease as the temperature decreases, which motivated us to redesign the amplitude control factor *F* so it can adaptively decrease as the number of iterations increases. The design principle and numerical analysis of the redesigned *F* indicate that the change in *F* could accelerate the convergence speed of the algorithm by improving the local exploitation capability. Furthermore, the redesigned *F* does not introduce extra parameters. We successfully implemented BSAISA to solve some constrained optimization and engineering design problems. The experimental results demonstrated that BSAISA has a faster convergence speed than BSA and it can efficiently balance the capacity for global exploration and local exploitation. The comparisons of the results obtained by BSAISA and other well-known algorithms demonstrated that BSAISA is more effective and competitive for constrained and engineering optimization problems in terms of convergence speed.

This paper suggests that the proposed BSAISA has a superiority in terms of convergence speed or computational cost. The downside of the proposed algorithm is, of course, that its robustness does not show enough superiority. So our future work is to further research into the robustness of BSAISA on the basis of current research. Niche technique is able to effectively maintain population diversity of evolutionary algorithms [82, 83]. How to combine BSAISA with niche technology to improve robustness of the algorithm may deserve to be studied in the future.

## Figures and Tables

**Pseudocode 1:**
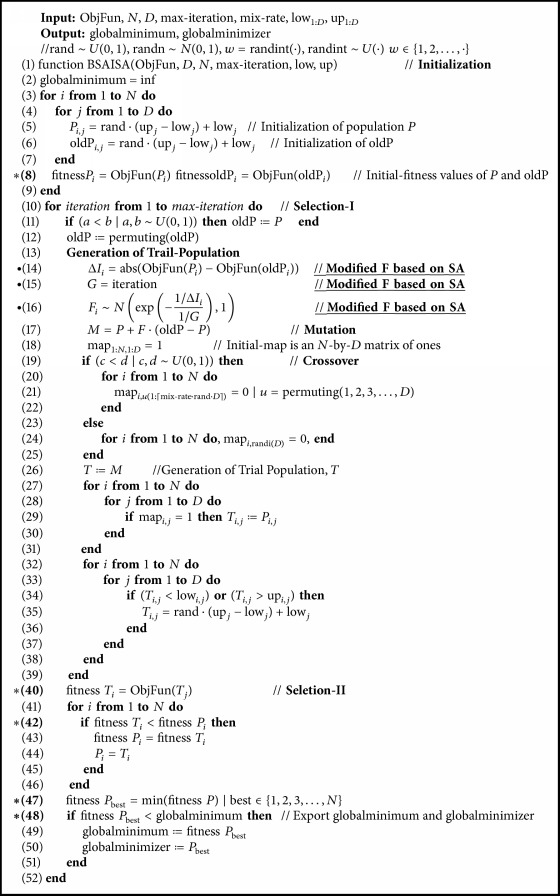
Pseudocode of BSAISA.

**Table 1:** Two benchmark functions and the corresponding populations, dimensions, and max iterations.

Name	Objective function	Range	*N*	*D*	Max
Schwefel 1.2	$f\left(x\right)=\overset{D}{\underset{i=1}{\sum }}{\left(\sum_{j=1}^{i}x_{j}\right)}^{2}$	[−100,100]	100	30	500
Rastrigin	$f\left(x\right)=\overset{D}{\underset{i=1}{\sum }}\left(x_{i}^{2}-10\cos\left(2\pi x_{i}\right)+10\right)$	[−5.12,5.12]	100	30	500

*Note*. “*N*” means populations, “*D*” means dimensions, and “Max” means maximum iterations.

**Table 2:** The parameter setting of SA*ε*.

*T*_*c*_ = 0.2*∗T*_max_	*θ* = 0.3*∗N*	cp = 5	Th1 = 10	Th2 = 2

*Note*. “*T*_max_” means the maximum iterations; “*N*” means populations.

**Table 3:** Characters of the 13 benchmark functions.

Fun.	*D*	Type	*ρ* (%)	LI	NI	LE	NE	Active
g01	13	Quadratic	0.0003	9	0	0	0	6
g02	20	Nonlinear	99.9973	1	1	0	0	1
g03	10	Nonlinear	0.0000	0	0	0	1	1
g04	5	Quadratic	27.0079	0	6	0	0	2
g05	4	Nonlinear	0.0000	2	0	0	3	3
g06	2	Nonlinear	0.0057	0	2	0	0	2
g07	10	Quadratic	0.0003	3	5	0	0	6
g08	2	Nonlinear	0.8581	0	2	0	0	0
g09	7	Nonlinear	0.5199	0	4	0	0	2
g10	8	Linear	0.0020	3	3	0	0	3
g11	2	Quadratic	0.0973	0	0	0	1	1
g12	3	Quadratic	4.7679	0	9^3^	0	0	0
g13	5	Nonlinear	0.0000	0	0	1	2	3

*Note*. “*D*” is the number of variables. “*ρ*” represents feasibility ratio. “LI,” “NI,” “LE,” and “NE” represent linear inequality, nonlinear inequality, linear equality, and nonlinear equality, respectively. “Active” represents the number of active constraints at the global optimum.

**Table 4:** User parameters used for all experiments.

Problem	G01–G13	TTP	PVP	TCSP	WBP	SRP
*N*	30	20	20	20	20	20
*T*_max_	11665	1000	3000	3000	3000	2000
Runs	30	50	50	50	50	50

*Note*. The expression “runs” denotes the number of independent runs.

**Table 5:** The statistical results of BSAISA for 13 constrained benchmarks.

Fun.	Known optimal	Best	Mean	Worst	Std	FEs
G01	−15	**−15**	−15	−15.000000	8.08*E* − 16	84,630
G02	−0.803619	−0.803599	−0.787688	−0.758565	1.14*E* − 02	349,500
G03	−1.000500	−1.000498	−1.000481	−1.000441	1.35*E* − 05	58,560
G04	−30665.538672	**−30665.538672**	−30665.538672	−30665.538672	1.09*E* − 11	121,650
G05	5126.496714	**5126.496714**	5126.496714	5126.496714	5.85*E* − 13	238,410
G06	−6961.813876	**−6961.813876**	−6961.813876	−6961.813876	1.85*E* − 12	89,550
G07	24.306209	24.307381	24.400881	24.758205	1.02*E* − 01	15,060
G08	−0.0958250	**−0.0958250**	−0.086683	−0.027263	2.37*E* − 02	30,930
G09	680.630057	**680.630057**	680.633025	680.680043	9.11*E* − 03	347,760
G10	7049.248021	7049.249056	7081.241789	7326.853581	6.24*E* + 01	346,980
G11	0.749900	**0.749900**	0.749900	0.749900	1.13*E* − 16	87,870
G12	−1	**−1**	−1	−1	0	5430
G13	0.0539415	**0.0539415**	0.1030000	0.4594033	9.80*E* − 02	349,800

*Note*. “Known optimal” denotes the best known function values in the literatures. “*Bold*” means the algorithm has found the best known function values. The same as Table 6.

**Table 6:** The statistical results of the basic BSA on 13 constrained benchmarks.

Fun.	Known optimal	Best	Mean	Worst	Std	FEs
G01	−15	**−15**	−15	−15.000000	6.60*E* − 16	99,300
G02	−0.803619	−0.803255	−0.792760	−0.749326	9.10*E* − 03	344,580
G03	−1.000500	−1.000488	−0.998905	−0.990600	2.66*E* − 03	348,960
G04	−30665.538672	**−30665.538672**	−30665.538672	−30665.538672	1.11*E* − 11	272,040
G05	5126.496714	**5126.496714**	5144.041363	5275.384724	3.99*E* + 01	299,220
G06	−6961.813876	**−6961.813876**	−6961.813876	−6961.813876	1.85*E* − 12	111,450
G07	24.306209	24.307607	24.344626	24.399896	1.93*E* − 02	347,250
G08	−0.0958250	**−0.0958250**	−0.0958250	−0.0958250	2.82*E* − 17	73,440
G09	680.630057	680.630058	680.630352	680.632400	5.63*E* − 04	348,900
G10	7049.248021	7049.278543	7053.573853	7080.192700	7.28*E* + 00	340,020
G11	0.749900	**0.749900**	0.749900	0.749900	1.13*E* − 16	113,250
G12	−1	**−1**	−1	−1	0	13,590
G13	0.0539415	0.0539420	0.1816986	0.5477657	1.50*E* − 01	347,400

**Table 7:** Comparison of the best values and FEs obtained by BSAISA and other algorithms.

Alg.	BSAISA	SR	FSA	CDE	AMA	MABC	RPGA	BSA-SA*ε*
Fun.	Best (FEs)	Best (FEs)	Best (FEs)	Best (FEs)	Best (FEs)	Best (FEs)	Best (FEs)	Best (FEs)
G01	**−15**	**−15.000**	−14.993316	**−15.000000**	**−15.000**	**−15.000**	**−15.000**	**−15.000000**
	(**84,630**)	(148,200)	(205,748)	(100,100)	(350,000)	(350,000)	(350,000)	(350,000)
G02	**−0.8036**	−0.8035	−0.7549	**−0.8036**	−0.8035	**−0.8036**	**−0.8036**	**−0.8036**
	(349,500)	(217,200)	(227,832)	(**100,100**)	(350,000)	(350,000)	(350,000)	(350,000)
G03	**−1.000498**	−1.000	−1.0000015	−0.995413	−1.000	−1.000	−1.000	**−1.000498**
	(**58,560**)	(229,200)	(314,938)	(100,100)	(350,000)	(350,000)	(350,000)	(350,000)
G04	**−30665.539**	**−30665.539**	−30665.538	**−30665.539**	−30665.538	**−30665.539**	**−30665.539**	**−30665.539**
	(121,650)	(**88,200**)	(86,154)	(100,100)	(350,000)	(350,000)	(350,000)	(350,000)
G05	5126.497	5126.497	5126.4981	5126.571	5126.512	**5126.487**	5126.544	5126.497
	(238,410)	(51,600)	(47,661)	(100,100)	(350,000)	(**350,000**)	(350,000)	(350,000)
G06	**−6961.814**	**−6961.814**	**−6961.814**	**−6961.814**	−6961.807	**−6961.814**	**−6961.814**	**−6961.814**
	(89,550)	(118,000)	(**44,538**)	(100,100)	(350,000)	(350,000)	(350,000)	(350,000)
G07	24.307	24.307	24.311	**24.306**	24.315	24.324	24.333	**24.306**
	(15,060)	(143,000)	(404,501)	(**100,100**)	(350,000)	(350,000)	(350,000)	(350,000)
G08	**−0.095825**	**−0.095825**	**−0.095825**	**−0.095825**	**−0.095825**	**−0.095825**	**−0.095825**	**−0.095825**
	(**30,930**)	(76,200)	(56,476)	(100,100)	(350,000)	(350,000)	(350,000)	(350,000)
G09	**680.630057**	680.630	680.63008	**680.630057**	680.645	680.631	680.631	680.6301
	(347,760)	(111,400)	(324,569)	(**100,100**)	(350,000)	(350,000)	(350,000)	(350,000)
G10	7049.249	7054.316	7059.864	**7049.248**	7281.957	7058.823	7049.861	7049.278
	(346,980)	(128,400)	(243,520)	(**100,100**)	(350,000)	(350,000)	(350,000)	(350,000)
G11	**0.749900**	0.750	0.749999	**0.749900**	0.750	0.750	0.749	**0.749900**
	(**87,870**)	(11,400)	(23,722)	(100,100)	(350,000)	(350,000)	(350,000)	(350,000)
G12	**−1**	**−1.000000**	**−1.000000**	**−1.000000**	**−1.000**	**−1.000**	NA	**−1.000000**
	(**5430**)	(16,400)	(59,355)	(100,100)	(350,000)	(350,000)		(350,000)
G13	**0.0539415**	0.053957	0.0539498	0.056180	0.053947	0.757	NA	**0.0539415**
	(**349,800**)	(69,800)	(120,268)	(100,100)	(350,000)	(350,000)		(350,000)
Nu.	6 + 10	1 + 5	1 + 3	4 + 10	0 + 3	1 + 7	0 + 5	0 + 10
RK	1	5	7	2	8	4	6	3

*Note*. “NA” means not available. The same as Tables 8, 10, 11, 12, 13, 14, 15, and 17. “RK” represents the comprehensive ranking of each algorithm on the set of benchmarks. “Nu.” represents the sum of the number of winners and the number of the optimal function values for each algorithm on the set of benchmarks.

**Table 8:** Comparison of best solutions for the three-bar truss design problem.

Method	DEDS	HEAA	PSO-DE	DELC	MBA	BSA	BSAISA
*X*_1_	0.788675	0.788680	0.788675	0.788675	0.788675	0.788675	0.788675
*X*_2_	0.408248	0.408234	0.408248	0.408248	0.408560	0.408248	0.408248
*g*_1_(*X*)	1.77*E* − 08	NA	−5.29*E* − 11	NA	−5.29*E* − 11	−3.23*E* − 12	0
*g*_2_(*X*)	−1.464102	NA	−1.463748	NA	−1.463748	−1.464102	−1.464102
*g*_3_(*X*)	−0.535898	NA	−0.536252	NA	−0.536252	−0.535898	−0.535898
*f*(*X*)	**263.895843**	**263.895843**	**263.895843**	**263.895843**	263.895852	**263.895843**	**263.895843**

**Table 9:** Comparison of statistical results for the three-bar truss design problem.

Method	Worst	Mean	Best	Std	FEs
DEDS	263.895849	263.895843	**263.895843**	9.7*E* − 07	15,000
HEAA	263.896099	263.895865	**263.895843**	4.9*E* − 05	15,000
PSO-DE	263.895843	263.895843	**263.895843**	4.5*E* − 10	17,600
DELC	263.895843	263.895843	**263.895843**	4.3*E* − 14	10,000
MBA	263.915983	263.897996	263.895852	3.93*E* − 03	13,280
BSA	263.895845	263.895843	**263.895843**	2.64*E* − 07	13,720
BSAISA	263.895843	263.895843	**263.895843**	5.75*E* − 13	**8940**

**Table 10:** Comparison of best solutions for the pressure vessel design problem.

Method	GA-DT	MDE	CPSO	HPSO	DELC	ABC	BSA-SA*ε*	BSA	BSAISA
*X*_1_	0.8125	0.8125	0.8125	0.8125	0.8125	0.8125	0.8125	0.8125	0.8125
*X*_2_	0.4375	0.4375	0.4375	0.4375	0.4375	0.4375	0.4375	0.4375	0.4375
*X*_3_	42.0974	42.0984	42.0913	42.0984	42.0984	42.0984	42.0984	42.0984	42.0984
*X*_4_	176.6540	176.6360	176.7465	176.6366	176.6366	176.6366	176.6366	176.6366	176.6366
*g*_1_(*X*)	−2.01*E* − 03	0	−1.37*E* − 06	NA	NA	0	−9.5*E* − 10	−3.69*E* − 08	0
*g*_2_(*X*)	−3.58*E* − 02	−0.035881	−3.59*E* − 04	NA	NA	−0.035881	−3.59*E* − 2	−0.035881	−0.035881
*g*_3_(*X*)	−24.7593	−0.0000	−118.7687	NA	NA	−0.000226	−1.2*E* − 4	−0.095446	0
*g*_4_(*X*)	−63.3460	−63.3639	−63.2535	NA	NA	−63.363	−63.363	−63.2842	−63.3634
*f*(*X*)	6059.9463	**6059.7017**	6061.0777	6059.7143	6059.7143	6059.7143	6059.7143	6059.7150	6059.7143

**Table 11:** Comparison of statistical results for the pressure vessel design problem.

Method	Worst	Mean	Best	Std	FEs
GA-DT	6469.3220	6177.2533	6059.9463	130.9297	80,000
MDE	6059.7017	6059.7017	**6059.7017**	1.0*E* − 12	24,000
CPSO	6363.8041	6147.1332	6061.0777	86.45	30,000
HPSO	6288.6770	6099.9323	6059.7143	86.20	81,000
DELC	6059.7143	6059.7143	6059.7143	2.1*E* − 11	30,000
PSO-DE	6059.7143	6059.7143	6059.7143	1.0*E* − 10	42,100
ABC	NA	6245.3081	6059.7147	2.05*E* + 02	30,000
BSA-SA*ε*	6116.7804	6074.3682	6059.7143	1.71*E* + 01	80,000
BSA	6771.5969	6221.2861	6059.7150	2.03*E* + 02	60,000
BSAISA	7198.0054	6418.1935	6059.7143	3.04*E* + 02	**16,320**

**Table 12:** Comparison of best solutions for the tension compression spring design problem.

Method	*X*_1_	*X*_2_	*X*_3_	*g*_1_(*X*)	*g*_2_(*X*)	*g*_3_(*X*)	*g*_4_(*X*)	*f*(*X*)
GA-DT	0.051989	0.363965	10.890522	−1.3*E* − 05	−2.1*E* − 05	−4.061338	−0.722698	0.012681
MDE	0.051688	0.356692	11.290483	−0.000000	−0.000000	−4.053734	−0.727747	**0.012665**
CPSO	0.051728	0.357644	11.244543	−8.45*E* − 04	−1.26*E* − 05	−4.051300	−0.727090	0.012675
HPSO	0.051706	0.357126	11.265083	NA	NA	NA	NA	**0.012665**
DEDS	0.051689	0.356718	11.288965	NA	NA	NA	NA	**0.012665**
HEAA	0.051690	0.356729	11.288294	NA	NA	NA	NA	**0.012665**
DELC	0.051689	0.356718	11.288966	NA	NA	NA	NA	**0.012665**
ABC	0.051749	0.358179	11.203763	−0.000000	−0.000000	−4.056663	−0.726713	**0.012665**
MBA	0.051656	0.35594	11.344665	0	0	−4.052248	−0.728268	**0.012665**
SSOC	0.051689	0.356718	11.288965	NA	NA	NA	NA	**0.012665**
BSA-SA*ε*	0.051989	0.356727	11.288425	−7.70*E* − 09	−3.30*E* − 09	−4.054	−0.728	**0.012665**
BSA	0.051694	0.356845	11.281488	−1.05*E* − 07	−1.77 − 08	−4.054037	−0.727640	**0.012665**
BSAISA	0.051687	0.356669	11.291824	−6.38*E* − 10	−1.53*E* − 09	−4.053689	−0.727763	**0.012665**

**Table 13:** Comparison of statistical results for the tension compression spring design problem.

Method	Worst	Mean	Best	Std	FEs
GA-DT	0.012973	0.012742	0.012681	5.90*E* − 05	80,000
MDE	0.012674	0.012666	**0.012665**	2.0*E* − 6	24,000
CPSO	0.012924	0.012730	0.012675	5.20*E* − 05	23,000
HPSO	0.012719	0.012707	**0.012665**	1.58*E* − 05	81,000
DEDS	0.012738	0.012669	**0.012665**	1.25*E* − 05	24,000
HEAA	0.012665	0.012665	**0.012665**	1.4*E* − 09	24,000
DELC	0.012666	0.012665	**0.012665**	1.3*E* − 07	20,000
PSO-DE	0.012665	0.012665	**0.012665**	1.2*E* − 08	24,950
ABC	NA	0.012709	**0.012665**	0.012813	30,000
MBA	0.012900	0.012713	**0.012665**	6.30*E* − 05	**7650**
SSOC	0.012868	0.012765	**0.012665**	9.29*E* − 05	25,000
BSA-SA*ε*	0.012666	0.012665	**0.012665**	1.62*E* − 07	80,000
BSA	0.012669	0.012666	**0.012665**	7.24*E* − 07	43,220
BSAISA	0.012668	0.012666	**0.012665**	4.90*E* − 07	9440

**Table 14:** Comparison of best solutions for the welded beam design problem.

Method	GA-DT	MDE	CPSO	HPSO	ABC	MBA	BSA-SA*ε*	BSA	BSAISA
*X*_1_(*h*)	0.205986	0.205730	0.202369	0.205730	0.205730	0.205729	0.205730	0.205730	0.205730
*X*_2_(*l*)	3.471328	3.470489	3.544214	3.470489	3.470489	3.470493	3.470489	3.470489	3.470489
*X*_3_(*t*)	9.020224	9.036624	9.04821	9.036624	9.036624	9.036626	9.036624	9.036624	9.036624
*X*_4_(*b*)	0.206480	0.205730	0.205723	0.205730	0.205730	0.205729	0.205730	0.205730	0.205730
*g*_1_(*X*)	−0.074092	−0.000335	−12.839796	NA	0.000000	−0.001614	−1.55*E* − 10	−5.32*E* − 07	0
*g*_2_(*X*)	−0.266227	−0.000753	−1.247467	NA	−0.000002	−0.016911	−4.30*E* − 09	−9.02*E* − 06	0
*g*_3_(*X*)	−4.95*E* − 04	−0.000000	−1.49*E* − 03	NA	0.000000	−2.40*E* − 07	−1.55*E* − 15	−7.86*E* − 12	−5.55*E* − 17
*g*_4_(*X*)	−3.430044	−3.432984	−3.429347	NA	−3.432984	−3.432982	−3.4330	−3.432984	−3.432984
*g*_5_(*X*)	−0.080986	−0.080730	−0.079381	NA	−0.080730	−0.080729	−8.07*E* − 02	−0.080730	−0.080730
*g*_6_(*X*)	−0.235514	−0.235540	−0.235536	NA	−0.235540	−0.235540	−0.2355	−0.235540	−0.235540
*g*_7_(*X*)	−58.666440	−0.000882	−11.681355	NA	0.000000	−0.001464	−1.85*E* − 10	−1.13*E* − 07	−5.46*E* − 12
*f*(*X*)	1.728226	**1.724852**	1.728024	**1.724852**	**1.724852**	1.724853	**1.724852**	**1.724852**	**1.724852**

**Table 15:** Comparison of statistical results for the welded beam design problem.

Method	Worst	Mean	Best	Std	FEs
GA-DT	1.993408	1.792654	1.728226	7.47*E* − 02	80,000
MDE	1.724854	1.724853	**1.724852**	NA	24,000
CPSO	1.782143	1.748831	1.728024	1.29*E* − 02	24,000
HPSO	1.814295	1.749040	**1.724852**	4.00*E* − 02	81,000
DELC	1.724852	1.724852	**1.724852**	4.1*E* − 13	**20,000**
PSO-DE	1.724852	1.724852	**1.724852**	6.7*E* − 16	66,600
ABC	NA	1.741913	**1.724852**	3.1*E* − 02	30,000
MBA	1.724853	1.724853	1.724853	6.94*E* − 19	47,340
SSOC	1.799332	1.746462	**1.724852**	2.57*E* − 2	25,000
BSA-SA*ε*	1.724852	1.724852	**1.724852**	8.11*E* − 10	80,000
BSA	1.724854	1.724852	**1.724852**	2.35*E* − 07	45,480
BSAISA	1.724854	1.724852	**1.724852**	2.96*E* − 07	29,000

**Table 16:** Comparison of best solutions for the speed reducer design problem.

Method	MDE	DEDS	DELC	HEAA	POS-DE	MBA	BSA	BSAISA
*X*_1_	3.500010	3.500000	3.500000	3.500023	3.500000	3.500000	3.500000	3.500000
*X*_2_	0.700000	0.700000	0.700000	0.700000	0.700000	0.700000	0.700000	0.700000
*X*_3_	17	17	17	17.000013	17.000000	17.000000	17	17
*X*_4_	7.300156	7.300000	7.300000	7.300428	7.300000	7.300033	7.300000	7.300000
*X*_5_	7.800027	7.715320	7.715320	7.715377	7.800000	7.715772	7.715320	7.715320
*X*_6_	3.350221	3.350215	3.350215	3.350231	3.350215	3.350218	3.350215	3.350215
*X*_7_	5.286685	5.286654	5.286654	5.286664	5.286683	5.286654	5.286654	5.286654
*f*(*X*)	2996.356689	**2994.471066**	**2994.471066**	2994.499107	2996.348167	2994.482453	**2994.471066**	**2994.471066**

**Table 17:** Comparison of statistical results for the speed reducer design problem.

Method	Worst	Mean	Best	Std	FEs
MDE	2996.390137	2996.367220	2996.356689	8.2*E* − 03	24,000
DEDS	2994.471066	2994.471066	**2994.471066**	3.58*E* − 12	30,000
HEAA	2994.752311	2994.613368	2994.499107	7.0*E* − 02	40,000
DELC	2994.471066	2994.471066	**2994.471066**	1.9*E* − 12	30,000
PSO-DE	2996.348204	2996.348174	2996.348167	6.4*E* − 06	54,350
ABC	NA	2997.058412	2997.058412	0	30,000
MBA	2999.652444	2996.769019	2994.482453	1.56	6300
SSOC	2996.113298	2996.113298	2996.113298	1.34*E* − 12	25,000
BSA	2994.471066	2994.471066	**2994.471066**	9.87*E* − 11	25,640
BSAISA	2994.471095	2994.471067	**2994.471066**	5.40*E* − 06	**15,860**

**Table 18:** Comparisons between BSAISA and other algorithms in Sign Tests.

BSAISA-methods	+	≈	−	Total	*p* value
SR	11	1	1	13	0.006
FSA	12	0	1	13	0.003
CDE	8	0	5	13	0.581
AMA	13	0	0	13	0.000
MABC	10	2	1	13	0.012
RPGA	11	0	0	11	0.001
BSA-SA*ε*	8	4	1	13	0.039

*Note*. The columns of “+,” “≈,” and “−” indicate the number of functions where BSAISA performs significantly better than, almost the same as, or significantly worse than the compared algorithm, respectively. “*p* value” denotes the probability value supporting the null hypothesis.

## Appendix

### A. Constrained Benchmark Problems

#### A.1. Constrained Problem 01

(A.1) min fx=5∑i=14xi−5∑i=14xi2−∑i=513xisubject  to: g1x=2x1+2x2+x10+x11−10≤0 g2x=2x1+2x3+x10+x12−10≤0 g3x=2x2+2x3+x11+x12−10≤0 g4x=−8x1+x10≤0 g5x=−8x2+x11≤0 g6x=−8x3+x13≤0 g7x=−2x4−x5+x10≤0 g8x=−2x6−x7+x11≤0 g9x=−2x8−x9+x12≤0 0≤xi≤1i=1,…,9 0≤xi≤100i=10,11,12 0≤x13≤1.

#### A.2. Constrained Problem 02

(A.2) max fx=∑i=1ncos4xi−2∏i=1ncos2xi∑i=1nixi2subject  to: g1x=0.75−∏i=1nxi≤0 g2x=∑i=1nxi−7.5n≤0 n=20,  0≤xi≤10,  i=1,…,n.

#### A.3. Constrained Problem 03

(A.3) max fx=nn·∏i=1nxisubject  to: hx=∑i=1nxi2=0 n=10,  0≤xi≤1,  i=1,…,n.

#### A.4. Constrained Problem 04

(A.4) min fx=5.3578547x32+0.8356891x1x5+37.293239x1−40792.141subject  to: g1x=85.334407+0.0056858x2x5+0.0006262x1x4−0.0022053x3x5−92≤0 g2x=−85.334407−0.0056858x2x5−0.0006262x1x4+0.0022053x3x5≤0 g3x=80.51249+0.0071317x2x5+0.0029955x1x2+0.0021813x32−110≤0 g4x=−80.51249−0.0071317x2x5−0.0029955x1x2−0.0021813x32+90≤0 g5x=9.300961+0.0047026x3x5+0.0012547x1x3+0.0019085x3x4−25≤0 g6x=−9.300961−0.0047026x3x5−0.0012547x1x3−0.0019085x3x4+20≤0 78≤x1≤102, 33≤x2≤45 27≤xi≤45,i=3,4,5.

#### A.5. Constrained Problem 05

(A.5) min fx=3x1+0.000001x13+2x2+0.0000023x23subject  to: g1x=−x4+x3−0.55≤0 g2x=−x3+x4−0.55≤0 h3x=1000sin⁡−x3−0.25+1000sin⁡−x4−0.25+894.8−x1=0 h4x=1000sin⁡x3−0.25+1000sin⁡x3−x4−0.25+894.8−x2=0 h5x=1000sin⁡x4−0.25+1000sin⁡x4−x3−0.25+1294.8=0 0≤x1,x2≤1200, −0.55≤x3,x4≤0.55.

#### A.6. Constrained Problem 06

(A.6) min fx=x1−103+x2−203subject  to: g1x=−x1−52−x2−52+100≤0 g2x=x1−62+x2−52−82.81≤0 13≤x1≤100, 0≤x2≤100.

#### A.7. Constrained Problem 07

(A.7) min fx=x12+x22+x1x2−14x1−16x2+x3−102+4x4−52+x5−32+2x6−12+5x72+7x8−112+2x9−102+x10−72+45subject  to: g1x=−105+4x1+5x2−3x7+9x8≤0 g2x=10x1−8x2−17x7+2x8≤0 g3x=−8x1+2x2+5x9−2x10−12≤0 g4x=3x1−22+4x2−32+2x32−7x4−120≤0 g5x=5x12+8x2+x3−62−2x4−40≤0 g6x=x12+2x2−22−2x1x2+14x5−6x6≤0 g7x=0.5x1−82+2x2−42+3x52−x6−30≤0 g8x=−3x1+6x2+12x9−82−7x10≤0 −10≤xi≤10,i=1,…,10.

#### A.8. Constrained Problem 08

(A.8) min fx=−sin32πx1sin⁡2πx2x13x1+x2subject  to: g1x=x12−x2+1≤0 g2x=1−x1+x2−42≤0 0≤xi≤10,i=1,2.

#### A.9. Constrained Problem 09

(A.9) min fx=x1−102+5x2−122+x34+3x4−112+10x56+7x62+x74−4x6x7−10x6−8x7subject  to: g1x=2x12+3x24+x3+4x42+5x5−127≤0 g2x=7x1+3x2+10x32+x4−x5−282≤0 g3x=23x1+x22+6x62−8x7−196≤0 g4x=4x12+x22−3x1x2+2x32+5x6−11x7≤0 −10≤xi≤10,i=1,2,3,4,5,6,7.

#### A.10. Constrained Problem 10

(A.10) min fx=x1+x2+x3subject  to: g1x=−1+0.0025x4+x6≤0 g2x=−1+0.0025x5+x7−x4≤0 g3x=−1+0.01x8−x5≤0 g4x=−x1x6+833.33252x4+100x1−83333.333≤0 g5x=−x2x7+1250x5+x2x4−1250x4≤0 g6x=−x3x8+1250000+x3x5−2500x5≤0 100≤x1≤10000 1000≤xi≤10000,i=2,3 10≤xi≤1000,i=4,5,6,7,8.

#### A.11. Constrained Problem 11

(A.11) min fx=x12+x2−12subject  to: hx=x2−x12=0 −1≤xi≤1,i=1,2.

#### A.12. Constrained Problem 12

(A.12) max fx=100−x1−52−x2−52−x3−522100subject  to: gx=x1−p2+x2−q2+x3−r2−0.0625≤0 p,q,r=1,…,9, 0≤xi≤10,i=1,2,3.

#### A.13. Constrained Problem 13

(A.13) min fx=ex1x2x3x4x5subject  to: h1x=x12+x22+x32+x42+x52−10=0 h2x=x2x3−5x4x5=0 h3x=x13+x23+1=0 −2.3≤xi≤2.3,i=1,2 −3.2≤xi≤3.2,i=3,4,5.

### B. Engineering Design Problems

#### B.1. Three-Bar Truss Design Problem

(B.1) min fx=22x1+x2×lsubject  to: g1x=2x1+x22x12+2x1x2P−σ≤0 g2x=x22x12+2x1x2P−σ≤0 g3x=12x2+x1P−σ≤0 0≤xi≤1,i=1,2 l=100 cm, P=2 kN/cm2, σ=2 kN/cm2.

#### B.2. Pressure Vessel Design Problem

(B.2) min fx=0.6224x1x3x4+1.7781x2x32+3.1661x12x4+19.84x12x3subject  to: g1x=−x1+0.0193x3≤0 g2x=−x2+0.00954x3≤0 g3x=−πx32x4−43πx33+1296000≤0 g4x=x4−240≤0 0≤xi≤100,i=1,2 10≤xi≤200,i=3,4.

#### B.3. Tension/Compression Spring Design Problem

(B.3) min fx=x3+2x2x12subject  to: g1x=−x23x371785x14+1≤0 g2x=4x22−x1x212566x2x13−x14+15108x12−1≤0 g3x=−140.45x1x22x3+1≤0 g4x=x1+x21.5−1≤0 0.05≤x1≤2.00 0.25≤x2≤1.30 2.00≤x3≤15.00.

#### B.4. Welded Beam Design Problem

(B.4) min fx=1.10471x12x2+0.04811x3x414+x2subject  to: g1x=τx−τmax≤0 g2x=σx−σmax≤0 g3x=x1−x4≤0 g4x=0.10471x12+0.04811x3x414+x2−5≤0 g5x=0.125−x1≤0 g6x=δx−δmax≤0 g7x=P−Pcx≤0 0.1≤xi≤2,i=1,4 0.1≤xi≤10,i=2,3,

where

(B.5) τx=τ′2+2τ′τ′′x22R+τ′′2,τ′=P2x1x2,  τ′′=MRJM=PL+x22,R=x224+x1+x322,J=22x1x2x2212+x1+x322σx=6PLx4x32,δx=4PL3Ex33x4,Pcx=4.013Ex32x46/36L2×1−x32LE4GP=6000 lb,L=14 in,E=30×106 psi,G=12×106 psiτmax=13600 psi,σmax=30000 psi,δmax=0.25 in.

#### B.5. Speed Reducer Design Problem

(B.6) min fx=0.7854x1x223.3333x32+14.9334x3−43.0934−1.508x1x62+x72+7.4777x63+x73+0.7854x4x62+x5x72subject  to: g1x=27x1x22x3−1≤0 g2x=397.5x1x22x32−1≤0 g3x=1.93x43x2x64x3−1≤0 g4x=1.93x53x2x74x3−1≤0 g5x=745x4/x2x32+16.9×1061/2110x63−1≤0 g6x=745x5/x2x32+157.5×1061/285x73−1≤0 g7x=x2x340−1≤0 g8x=5x2x1−1≤0 g9x=x112x2−1≤0 g10x=1.5x6+1.9x4−1≤0 g11x=1.1x7+1.9x5−1≤0,

where

(B.7) 2.6≤x1≤3.6,0.7≤x2≤0.8,17≤x3≤287.3≤x4,x5≤8.3,2.9≤x6≤3.9,5.0≤x7≤5.5.
